# Domain-specific brain regions are associated with cognitive impairment in progressive supranuclear palsy

**DOI:** 10.1016/j.ynirp.2025.100247

**Published:** 2025-03-04

**Authors:** N. Schröter, M. Rijntjes, J.A. Hosp, M. Reisert, H. Mast, C. Weiller, P. Oikonomou, L. Frings, H. Urbach, W.H. Jost, A. Rau

**Affiliations:** a Department of Neurology and Clinical Neuroscience, Medical Center – University of Freiburg, Faculty of Medicine, University of Freiburg, Freiburg, Germany; b Medical Physics, Department of Radiology, Medical Center – University of Freiburg, Faculty of Medicine, University of Freiburg, Germany; c Department of Stereotactic and Functional Neurosurgery, Medical Center – University of Freiburg, Faculty of Medicine, University of Freiburg, Freiburg, Germany; d Department of Neuroradiology, Medical Center – University of Freiburg, Faculty of Medicine, University of Freiburg, Freiburg, Germany; e Parkinson-Klinik Ortenau, Wolfach, Germany; f Department of Nuclear Medicine, Medical Center – University of Freiburg, Faculty of Medicine, University of Freiburg, Freiburg, Germany

**Keywords:** Progressive supranuclear palsy, Cognitive deficits, Diffusion multicompartment imaging, Diffusion microstructure imaging, Frontal lobe dysfunction

## Abstract

**Background:**

Cognitive impairment significantly contributes to the disease burden of progressive supranuclear palsy (PSP), however, the underlying pathophysiologiy is not well understood.

**Objectives:**

To gain a better understanding of the pathophysiology, we identified the brain regions associated with individual domains of impaired cognition.

**Methods:**

We analyzed MRI data from a cohort of 31 patients with PSP (age 71.0 +-7.0 years, range 58–87; 15 females; disease duration 2.9 +- 1.8 years). Cerebral microstructure was approximated with Diffusion Microstructure Imaging and cognitive performance was measured using the Frontal Assessment Battery (FAB) and Montreal Cognitive Assessment (MoCA). To reveal the underlying affected brain regions, whole-brain voxel-wise associations were employed to test the microstructural metrics regarding their correlation with the FAB as well as the individual cognitive domains ‚Attention‘, ‚Execution‘, ‚Language‘, ‚Memory‘, ‚Orientation‘, and ‚Visuoconstruction‘ derived from MoCA.

**Results:**

MoCA performance was impaired in 87.5% of patients (20.2 +- 5.4 points, range 8–28; cut-off value: <26/30). In the voxel-wise analyses, we noted significant associations of cerebral microstructure and FAB in the right-sided frontal and temporopolar white matter, deficits in ‚Memory‘ with hippocampal and temporomesial regions, in reduced ‚Orientation‘ with wide spread white-matter areas with a parietal accentuation, whereas deficits in ‚Attention‘ correlated with frontal and prefrontal structures.

**Conclusions:**

Diffusion Microstructure Imaging revealed domain-specific regions of neurodegenerative alterations in PSP. The regions identified in this approach integrate well in existing disease concepts. They might therefore be a possible biomarker for cognitive impairment, as well as amonitoring parameter for future disease modifying therapeutics.

## Highlights

• Different cognitive domains are affected in progressive supranuclear palsy.
• MRI-based Diffusion Microstructure Imaging revealed neuroanatomical correlates.
• Memory deficits were linked to hippocampal and temporomesial degeneration.
• Attention deficits were associated with fronto-temporal white matter alterations.
• Orientation deficits correlated with widespread white matter, especially in parietal regions.

## 1 Introduction

Alongside ocular motor dysfunction, akinesia and postural instability, cognitive dysfunction constitutes a diagnostic hallmark of progressive supranuclear palsy (PSP), significantly contributing to disease burden in both patients and caregivers (Höglinger et al., 2017; Schrag et al., 2003). Cognitive impairment is present in more than half of PSP patients and ranges from subjective cognitive impairment to dementia (Brown et al., 2010; Lee et al., 2023; Pilotto et al., 2017). Here, especially fronto-executive impairment is frequently present and comprises difficulty with planning and organization, often accompanied by apathy and dysfunction of inhibition (Gerstenecker et al., 2013). Moreover, patients suffer from reduced verbal fluency (Bak et al., 2005; Grafman et al., 1990; Soliveri et al., 2000), attention, processing speed and memory (Brown et al., 2010; Burrell et al., 2014; Horta-Barba et al., 2021).

The neuropathological basis of PSP is characterized by the accumulation of intracellular four-repeat tau protein affecting both neurons and glial cells (Jellinger, 2023). These deposits are predominantly found in subcortical regions (including the globus pallidus, subthalamic nucleus, and the dentate nucleus) as well as the cortex such as in limbic areas, precentral and angular gyrus (Brown et al., 2010; Hof et al., 1992; Jellinger, 2008).

Earlier studies identified neurodegeneration in the frontotemporal regions as well as in the basal ganglia, midbrain, and cerebellum in PSP. The extent of this atrophy was associated with a higher tau burden (Nicastro et al., 2020). Moreover, both cortical neurodegeneration and tau burden were associated with cognitive deficits which underlines the pathophysiological relevance of cortical involvement (Bigio et al., 1999; Koga et al., 2017).

Though a large proportion of patients with PSP develop cognitive deficits, some do not and time to conversion from mild cognitive impairment to dementia differs between patients (Pilotto et al., 2017). Therefore, the early identification of patients at high risk for cognitive deficits or cognitive deterioration is imperative, as compared to motor deficits, these lead to different limitations in daily functioning and necessitate separate approaches regarding therapies, life-planning and psychosocial support (Dröes et al., 2011; Morley et al., 2015; Müller, 2016, p. 201; Rittman et al., 2016).

Using MRI, macroscopic atrophy holds significant diagnostic value due to its detectability through widely available methods and simple assessment (Illán-Gala et al., 2022). As microstructural alterations precede macrostructural atrophy, advanced MRI techniques could allow for identifying neurodegeneration in regions linked to cognitive function before they are detectable in conventional imaging (Bai et al., 2022; Zhuang et al., 2013).

The cerebral microstructural composition can be non-invasively approximated by diffusion microstructure imaging (DMI). DMI is a biophysically motivated approach that mesoscopically distinguishes between different anatomical compartments based on their diffusion properties and was applied in several neurodegenerative Parkinson syndromes (Rau et al., 2022a, 2024; Reisert et al., 2017; Schröter et al., 2022, 2024).

In this study, we investigated the pathophysiological significance of cerebral microstructural integrity for specific neuropsychological deficits in PSP. We hypothesize that: (1) The examination of cerebral microstructure is more sensitive than the examination of cerebral macrostructure to investigate the association with neuropsychological deficits and that (2) deficits in frontal lobe function and the individual neurocognitive domains ‚Attention‘, ‚Execution‘, ‚Language‘, ‚Memory‘, ‚Orientation‘, and ‚Visuoconstruction‘ are associated with specific microstructural changes in distinct neuroanatomical regions.

## 2 Methods

### 2.1 Participants

This retrospective analysis included DMI data from consecutive patients with PSP who received routine MRI in a 3-year period between January 2020 and December 2022 for the differential diagnosis of atypical Parkinson syndromes. The inclusion criteria were presence of probable PSP, availability of German version of the Montreal Cognitive Assessment (MoCA) and/or frontal assessment battery (FAB) performed within 21 days of imaging, and a diffusion MRI (dMRI) conducted. Clinical diagnoses were validated by two board-certified neurologists (WJ, NSc) based on current diagnostic criteria (Höglinger et al., 2017); all available medical records were used for the consensus diagnosis. Imaging data indicative of PSP were available in the form of 18-Fluorodeoxyglucose (FDG)-PET data in all patients.

### 2.2 Neuropsychological testing

Cognitive functions were assessed with the MoCA (version 7.1, www.mocatest.org (Nasreddine et al., 2005);). The highest possible global MoCA score is 30 with higher scores indicating better performance. As suggested in the original publication, the cut-off score for cognitive impairment was defined as < 26 (Nasreddine et al., 2005). Correction for years of education (YoE) was performed for the global MoCA score (+1 point in case of ≤12 YoE). As described bei Nasreddine, MoCA comprises subtests in the six cognitive domains orientation (spatial and temporal orientation), attention (digit span, letter A tapping and subtraction), executive function (trail making, abstraction, and word fluency), visuoconstructive function (cube copying and clock drawing), language (naming, sentence repetition), and memory (delayed word recall). Domain scores were calculated as the sum of the subtest scores. We employed the FAB to assess frontal lobe dysfunction, the maximum score is 18 points with higher scores indicating better performance (Dubois et al., 2000).

### 2.3 Imaging acquisition and analysis

MRI acquisition, normalization, and calculation of DMI parameters was performed as previously described (Schröter et al., 2022). In brief, preoperative 3 T MRIs (MAGNETOM Prisma, Siemens Healthcare, Erlangen, Germany) comprising high resolution isotropic 3D-T1w imaging and multishell dMRI data were transferred to a local instance of the postprocessing platform NORA (www.nora-imaging.org) for further analysis. Following pre-processing of the diffusion-weighted images, we estimated microstructural diffusion metrics based on a three-compartment diffusion model using a Bayesian approach. We determined (I) the free water/CSF fraction (V-CSF), (II) the volume fraction within neurites (V-intra) and (III) the volume fraction outside the neurites (V-extra) (Reisert et al., 2017; Schröter et al., 2022). T1w imaging datasets were segmented into white matter, gray matter and cerebrospinal fluid (CSF) using CAT12 (http://www.neuro.uni-jena.de/cat/). DMI images were affinely co-registered to the T1w images and validity of co-registration was manually confirmed. Quality control further involved visually inspecting each individual DMI dataset and CAT12 segmentation. The diffeomorphic warp (obtained from normalization of T1w data) was subsequently used to transfer the quantitative dMRI maps to the Montreal Neurological Institute (MNI) space. Images were smoothed with a 3 mm full-width at half-maximum (FWHM) Gaussian kernel.

In previous studies, V-CSF has been shown to be a good surrogate marker for the extent of neurodegeneration in both gray and white matter (Rau et al., 2024; Schröter et al., 2022). To test for associations between MoCA subdomains and FAB with DMI parameters, voxel-based analyses were performed using SPM, where V-CSF was used as a dependent variable and threshold-free cluster enhancement (https://github.com/markallenthornton/MatlabTFCE) was applied as implemented in the Statistical Parametric Mapping-Voxel-Based Morphometry (SPM-VBM) 8-Toolbox. Here, age and sex served as nuisance covariates and FWE-correction was applied to control for multiple comparisons.

### 2.4 Demographic statistical analyses

Statistical analyses were performed using R (version 4.1.2, https://www.R-project.org/). Data are presented as the mean and standard deviation for continuous variables, and as absolute frequencies and percentages for categorical variables.

### 2.5 Data and code availability

Data and code are available from the corresponding author upon reasonable request and

approval by the local ethics committee. The approval of the local ethics committee is

obtained on a case-by-case basis for each request by the corresponding author.

## 3 Results

### 3.1 Patient characteristics

We report MRI and clinical data of 31 patients with PSP (mean age 71.0 +- 7.0 years, range 58–87 years; 15 female; 19 PSP-parkinsonism (PSP-P), 9 PSP-Richardson syndrome, (PSP-RS), 2 PSP-frontal (PSP-F), 1 PSP-corticobasal syndrome (PSP-CBS)). MoCA was available in all 31 patients, FAB in 29 patients. Cognitive impairment was found in 25/31 patients who performed below the cutoff of <26 points in the MoCA. MoCA domain scores revealed impairment especially in visuoconstruction, executive function and memory, while orientation and language were mostly preserved. Frontal lobe dysfunction as measured by FAB was present in 14/29 patients performing below the cutoff of≤12 points (Slachevsky et al., 2004). Detailed patient characteristics and data of neuropsychological testing are presented in Table 1.

**Table 1:** Patient characteristics.

Demographic data (mean ± SD, [range])	
Age (years)	71.0 ± 7.0 [58–87]
Sex (male/female)	16/15
Disease duration (years)	2.9 ± 1.8 [1.0–8.0]
UPDRS Total	62.1 ± 20.3 [21–110]
UPDRS Part III	36.6 ± 12.4 [9–67]

**Global cognitive Performance (mean ± SD, [range])**	

FAB (max. 18) Available in n = 29	12.2 ± 3.3 [5–18]
MoCA (max. 30)	20.8 ± 5.4 [8–30]

**MoCA Domain Scores (median [IQR])**	

Visuoconstructive (max. 4)	3 [2–3]
Language (max. 5)	5 [4–5]
Executive (max. 4)	1 [1–2]
Attention (max. 6)	5 [4–6]
Memory (max. 5)	3 [1–3.5]
Orientation (max. 6)	6 [5–6]

*UPDRS III*, Unified Parkinson's Disease Rating Scale; *FAB,* Frontal Assessment Battery*; MoCA,* Montreal Cognitive Assessment*; SD* standard deviation; *IQR* interquartile range.

### 3.2 Gray matter voxel based morphometry is not associated with cognitive dysfunction

To investigate the potential of conventional, structural MRI in providing insight into affected regions in cognitive decline in PSP, we carried our voxel-wise whole brain associations of scores of cognitive performance with CAT12-derived tissue probability values (TPV). Here, no significant association was observed between TPV and FAB as well as TPV and MoCA sumscore or MoCA-derived cognitive domains.

### 3.3 Microstructural alterations associated with impairment in cognitive subdomains

**Fig. 1:**
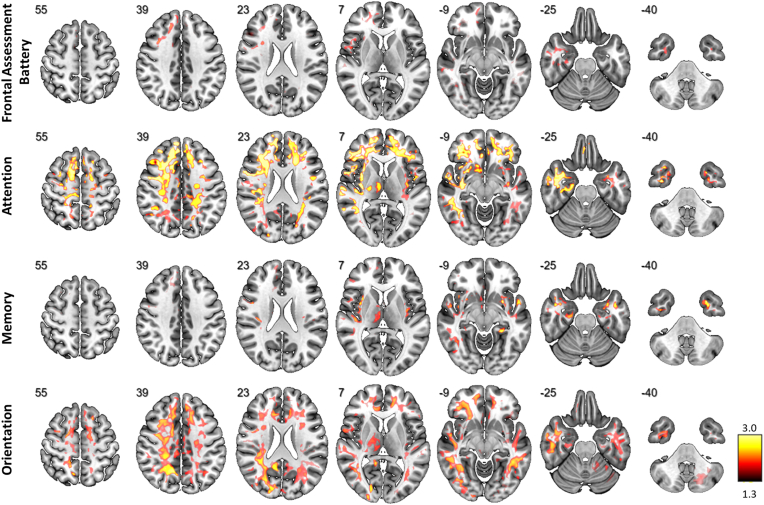
Significant association between V-CSF and cognitive performance as measured with the Frontal Assessment Battery (top row), and Montreal Cognitive Assessment attention (second row), memory (third row) and orientation (bottom row) subdomains. To relate clinical outcomes to V-CSF (microstructural free-fluid fraction) in patients with PSP, voxel-based analyses were performed, with V-CSF as dependent variable, threshold-free cluster enhancement and nuisance covariates “age” and “sex”. P-values were corrected for multiple comparisons across voxels using the family-wise error rate (FWE). Voxels with significantly different V-CSF are indicated by hot shading and superimposed onto a T1w MRI template (p-values are log-transformed with a lower threshold of 1.3; higher significance is given in more yellow color). Radiological orientation, i.e. left side of the image corresponds to the right side of the patient's body; numbers denote the axial (*z*) position in millimeters.

Frontal lobe dysfunction as measured by FAB was associated with an increased free-fluid fraction in the right-sided frontal and temporopolar white matter (Fig. 1).

**Fig. 2:**
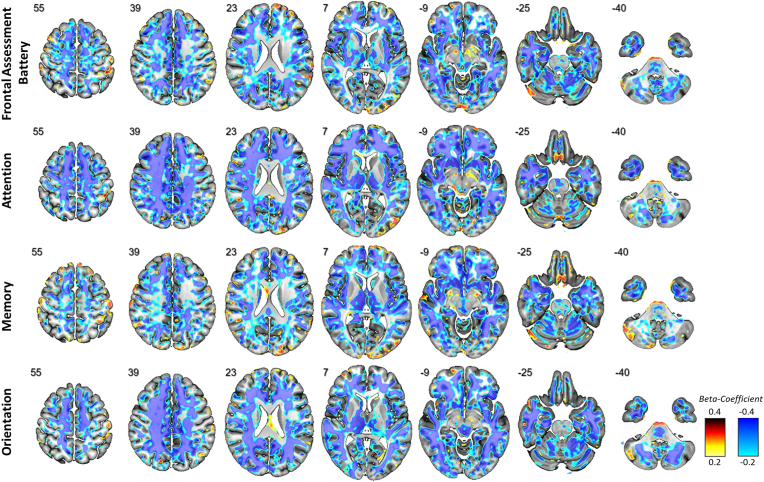
Standardized beta-coefficients were extracted from voxel-based analyses of V-CSF V-CSF and cognitive performance as measured with the Frontal Assessment Battery (top row), and Montreal Cognitive Assessment attention (second row), memory (third row) and orientation (bottom row) subdomains. Results are superimposed onto a T1w MRI template (positive effect directions are given in hot and negative in cold). Radiological orientation, i.e. left side of the image corresponds to the right side of the patient's body; numbers denote the axial (*z*) position in millimeters.

For the respective subdomains of the MoCA test, we noted distinct regional patterns of microstructural alterations. In detail, reduced performance in the attention-subdomain was associated with increased free-fluid fraction, in widespread white matter regions with a fronto-temporal emphasis (Fig. 1). Lower scores in the memory subdomain were associated with primarily cortically elevated free interstitial fluid in bilateral temporomesial regions including the hippocampus and also in the right medial thalamus (Fig. 1). Voxel-wise associations of the orientation subdomain revealed significant voxels in widespread white-matter areas with a parietal accentuation (Fig. 1). Standardized beta-coefficients are given in Fig. 2 to allow a detailed investigation of the effect sizes and directions.

No significant voxel-wise associations with DMI metrics were noted for the subdomains language, visuocontruction, and execution.

## 4 Discussion

In patients with early stage PSP, DMI revealed that neurodegeneration in specific neuroanatomical regions was strongly associated with cognitive impairment. We observed that (1) while DMI showed a strong association between cerebral microstructure and cognitive deficits, no such association was detectable for macroscopic atrophy, suggesting a higher sensitivity of DMI over conventional volumetric approaches as a biomarker of disease severity for neuropsychological deficits; and (2) associating the cerebral microstructure with impaired cognition in patients with PSP revealed symptom-specific regions for various domains of cognition. While frontal lobe dysfunction as measured by FAB was associated with pronounced neurodegeneration in right frontal and temporopolar white matter, attention was associated with widespread subcortical neurodegeneration with a fronto-temporal emphasis. Memory was associated with cortical limbic neurodegeneration, especially in the hippocampus, and orientation correlated with widespread white-matter degeneration with a parietal accentuation.

Noteworthy, our whole-brain approach without *a priori* assumptions regarding the spatial pattern revealed associations between different cognitive domains and brain regions that were previously linked to the respective function.

In detail, frontal lobe dysfunction as measured with FAB performance was primarily linked to the frontal white matter. This was corroborated by the regions unveiled by voxel-wise analyses of the attention MoCA subdomain. Here, attention deficits were associated with impaired microstructure in widespread fronto-temporal white matter regions which aligns with the central role the frontal cortex plays in attention in general (Daffner et al., 2000; Mesulam, 1990; Stuss, 2011; Weiller et al., 2021). Of note, almost all patients had severely impaired executive function as is common in PSP (Gerstenecker et al., 2013; O'Keeffe et al., 2007). This might explain the fact that we did not observe an association between microstructural metrics and impaired executive function as the variance to detect such an association was minimal.

Impaired episodic memory has repeatedly been described in PSP, however the underlying pathology is still unclear (Macedo et al., 2022). We observed a strong correlation between memory impairment with elevated interstitial free fluid in temporomesial regions and the right medial thalamus. Our findings are in line with the fact that the role of the mesiotemporal lobe in memory formation is well-established in both healthy individuals and neurodegeneration (Squire and Zola-Morgan, 1991).

Visuospatial deficits have been described in PSP and mainly be attributed to oculomotor dysfunction and overall cognitive decline (Bak et al., 2006). This interpretation is supported by our data, as no cerebral microstructural correlate was found for the observed mild visuoconstructive deficits.

Previous research suggests involvement of frontal, parietal, and temporal regions in spatial, temporal, and situational orientation (Bak et al., 2006). Consistent with this, we observed significant associations of comparable spatial distribution with the domain of ‚orientation‘, primarily composed of the three components of spatial, temporal, and situational orientation within the MoCA. Recent studies also point to a connection between orientation and the default mode network which is known to be altered in PSP and encompasses the aforementioned regions (Bharti et al., 2017).

The feasibility of the MoCA test to identify impaired cognition in PSP in a cross-sectional setting was described before (Pereira et al., 2022) supporting the choice of the MoCA to assess different cognitive domains in our study. Here, 25/31 patients showed impaired cognition as they performed below the cut-off of the MoCA. Moreover, the profile of impairment in distinct cognitive domains well fits previously identified patterns (Duff et al., 2019).

Our findings are particularly intriguing in the context of studies for disease-modifying therapies, as they suggest that the focus as an outcome measure should not only be on the midbrain and frontal lobes but should also encompass other strategically important regions like the parietal and temporal lobe (Dam et al., 2021; Höglinger et al., 2021).

The retrospective nature of this study constitutes a limitation of this study. Furthermore, post-mortem diagnoses were lacking. However, clinical diagnoses were validated by two experts in movement disorders. Additionally, FDG-PET supportive for the diagnosis of PSP was available in all patients (Meyer et al., 2016). Of note, the heterogeneity of subtypes of PSP is only represented to a limited extent in our cohort. However, since we enrolled two of the most common variants of PSP, i.e. PSP-RS and PSP-P, in relevant numbers in our cohort and it has been shown that there is a shift in the variants towards PSP-RS during the course of the disease, we assume that our data can be generalized for the majority of PSP patients (Sánchez-Ruiz de Gordoa et al., 2022; Street et al., 2021). Moreover, a comprehensive neuropsychological assessment would allow for a more detailed resolution of impairment in cognitive subdomains (Hendershott et al., 2017). Lastly, we relied on DMI only and did not investigate diffusion tensor imaging or neurite orientation and dispersion imaging (NODDI). However, previous research provided evidence that the Bayesian approach in DMI calculation allows for a better approximation of the microstructural composition in pathologically altered tissue (Rau et al., 2022a, 2022b; Reisert et al., 2017; Schröter et al., 2024; Würtemberger et al., 2023).

Future research should continue to explore the longitudinal trajectory of cognitive decline and potential therapeutic interventions for managing cognitive impairment in PSP. Especially against the background of distinct therapeutic regimes of different cognitive dysfunctions, further research should aim to validate these non-invasive biomarkers for optimized decision-making and therapy monitoring.

In conclusion, the regional specificity of altered cerebral microstructural associations with neurocognitive impairment uncovers cognitive domain-specific regions in PSP. This underscores the potential for advanced imaging techniques to provide valuable insights into the pathophysiology of cognitive impairment in PSP and may provide new biomarkers for diagnosis and decision-making.

## CRediT authorship contribution statement

**N. Schröter:** Writing – review & editing, Writing – original draft, Visualization, Validation, Methodology, Investigation, Funding acquisition, Formal analysis, Data curation, Conceptualization. **M. Rijntjes:** Writing – review & editing, Supervision, Methodology, Data curation. **J.A. Hosp:** Writing – review & editing, Methodology, Investigation. **M. Reisert:** Writing – review & editing, Validation, Supervision, Software. **H. Mast:** Writing – review & editing, Data curation. **C. Weiller:** Writing – review & editing, Validation, Supervision. **P. Oikonomou:** Writing – review & editing, Data curation. **L. Frings:** Writing – review & editing, Validation, Methodology. **H. Urbach:** Writing – review & editing, Supervision, Software. **W.H. Jost:** Writing – review & editing, Validation, Supervision, Data curation. **A. Rau:** Writing – review & editing, Writing – original draft, Visualization, Validation, Supervision, Methodology, Investigation, Formal analysis, Data curation, Conceptualization.

## Ethical compliance statement

The study was approved by the Institutional Review Board (Ethics Committee—University of Freiburg, EK 400/20) and carried out in accordance with the Declaration of Helsinki and its later amendments. Due to the retrospective nature of this study, the need for written informed consent was waived. We confirm that we have read the Journal's position on issues involved in ethical publication and affirm that this work is consistent with those guidelines.

## Declaration of competing interest

The authors declare that there are no conflicts of interest relevant to this work.

## Funding sources

No specific funding was received for this work.

## Financial disclosures for the previous 12 months

NS received honoraria from STADAPHARM. HU received honoraria from Eisai, Biogen, Lilly, mbits, Bayer. AR received honoraria from Bayer.

## Declaration of competing interest

The authors declare that they have no known competing financial interests or personal relationships that could have appeared to influence the work reported in this paper.

## Data Availability

Data will be made available on request.
